# Fibroblast growth factor 21 and its relationship with central precocious puberty in girls

**DOI:** 10.1530/EC-26-0320

**Published:** 2026-08-27

**Authors:** Xiaojing Hao, Jiangya Wang, Ran Zhou, Meng Sun, Yaying Cheng

**Affiliations:** ^1^ Department of Pediatrics, Hebei Medical University, Shijiazhuang, China; ^2^ Department of Pediatrics, Hebei General Hospital, Shijiazhuang, China

**Keywords:** central precocious puberty, girl, FGF21, β-Klotho

## Abstract

**Background:**

Studies have shown that obesity and insulin resistance are associated with central precocious puberty (CPP) in girls. Serum fibroblast growth factor 21 (FGF21) levels are associated with obesity and insulin resistance and are also related to ovarian development, reproductive function, and gonadotropin-releasing hormone (GnRH) neuron development. The aim of this study was to investigate the levels of serum FGF21 in girls with CPP.

**Methods:**

This study enrolled 106 girls aged 6–10 years who were evaluated at the hospital’s Growth and Development Clinic. The cohort comprised 55 girls diagnosed with CPP as the case group and 51 age-matched girls with normal growth and development as the control group. Data including age, age at onset of puberty or menarche, anthropometric measurements, IGF1 levels, bone age (BA), ovarian and uterine development, hormone profiles, β-Klotho levels, and FGF21 levels were collected and statistically analyzed.

**Results:**

Bone age advancement, IGF1, β-Klotho, and FGF21 levels showed statistically significant differences between the CPP and control groups. FGF21 and β-Klotho levels were negatively associated with CPP, with areas under the ROC curve of 0.833 and 0.770, respectively. The optimal cut-off values were 156.28 pg/mL and 7,057.18 pg/mL for β-Klotho. In girls with CPP, the FGF21 levels were weakly positively correlated with the β-Klotho levels (*r* = 0.273).

**Conclusion:**

Compared with age-matched girls without pubertal initiation, girls diagnosed with CPP had significantly lower serum levels of FGF21 and β-Klotho, suggesting that FGF21 may be involved in the regulation of pubertal onset. However, the underlying molecular mechanisms require further investigation.

## Background

The global incidence of precocious puberty has shown a steady increase in recent years (1, 2). Evidence suggests that the pathogenesis of central precocious puberty (CPP) involves the converging contributions of genetic susceptibility, exposure to environmental endocrine disruptors, and nutritional/metabolic imbalances (3). The implications of precocious puberty extend beyond the immediate consequences of reduced final height and psychological distress to encompass an elevated risk of chronic conditions in adulthood, including cardiovascular disease and breast cancer (4).

Reproductive hormones, including gonadotropin-releasing hormone (GnRH), luteinizing hormone (LH), follicle-stimulating hormone (FSH), and estrogen, play pivotal roles in maintaining the normal function of numerous reproductive processes. The central regulator of this system is GnRH, which is synthesized and released from the anterior and mediobasal hypothalamus (5). GnRH is secreted in a pulsatile manner and acts on gonadotroph cells in the anterior pituitary, thereby stimulating the release of gonadotropins (LH and FSH), which in turn promote gametogenesis and sex steroid production in the gonads (6). Premature elevation in the synthesis and secretion of GnRH can lead to the development of CPP (3), which is characterized by the emergence of secondary sexual characteristics before 8 years of age in females and 9 years of age in males, with a distinct female predominance.

FGF21 was initially identified as a rapid-response hormone and signals to multiple target tissues, such as the brain (7), adipose tissue (8), and pancreas (9), via a receptor complex composed of fibroblast growth factor receptor 1c (FGFR1c) and the co-receptor β-Klotho (10). Both FGFR1c and β-Klotho are expressed in hypothalamic neurons (11), and FGF21 has been shown to cross the blood–brain barrier in a non-saturable, unidirectional manner (12, 13). In addition, β-Klotho knockout mice exhibit delayed puberty, disrupted estrous cyclicity, and subfertility accompanied by impaired GnRH function (11). These findings suggest that the hepatokine FGF21, a key central metabolic regulator, may play an important role in linking metabolic signals to reproductive outcomes.

The reproductive process in mammals is complex, exhibiting high plasticity and flexibility through finely tuned mechanisms. These mechanisms depend on coordinated interactions among hypothalamus, pituitary, and gonadal tissues, commonly referred to as the ‘reproductive axis’ or hypothalamic–pituitary–gonadal axis (HPGA) (14, 15). Consequently, reproductive function is regulated by a variety of factors within the hypothalamic, pituitary, and ovarian tissues. Metabolism is recognized as an important modulator of HPGA function (16). Fibroblast growth factor (FGF) signaling plays a critical role in olfactory bulb morphogenesis, as well as in the specification, migration, and maturation of GnRH neurons. As a member of the endocrine FGF family, FGF21 is mainly synthesized in the liver. Previous research has found that FGF21 acts through the co-receptors β-Klotho and FGF receptor 1 to exert regulatory effects on the body through the central nervous system (17). Animal studies (18) have revealed that prenatal exposure of pregnant mice to the environmental contaminant perfluorononanoic acid increases hepatic synthesis and release of FGF21 in female offspring. Elevated FGF21 levels subsequently impair excitatory drive onto kisspeptin neurons, ultimately delaying pubertal activation of the GnRH–LH axis, resulting in delayed puberty. Accumulating evidence indicates that energy metabolism plays a crucial role in initiating puberty in mammals (19, 20). Notably, FGF21 is a key regulator of energy homeostasis (17, 21). Therefore, we hypothesize that FGF21 plays an important role in regulating GnRH release, contributing to the pathogenesis of CPP.

Current research on FGF21 predominantly focuses on adult diseases, with limited investigations conducted in pediatric populations. Although the regulatory role of FGF21 in the reproductive system has been validated (7), only a small number of studies have explored its association with pubertal onset, and clinical evidence regarding the relationship between FGF21 and CPP remains scarce. Accordingly, this study assessed serum FGF21 levels in girls with CPP and healthy girls with normal pubertal development to explore the potential role of FGF21 in pubertal onset.

## Methods

### Study sample and groups

This study enrolled 106 girls who were evaluated at the hospital’s Growth and Development Clinic between June 2024 and October 2025. The cohort comprised 55 girls diagnosed with CPP as the case group and 51 age-matched girls with normal growth and development as the control group. The CPP diagnosis was established according to the ‘Expert consensus on the diagnosis and treatment of central precocious puberty (2022)’ (22) requiring the presence of breast development (Tanner stage B2+) before 7.5 years of age or menarche before 10.0 years of age, bone age advancement of ≥1 year relative to chronological age, a peak LH level of ≥5I U/L and a peak LH/FSH ratio of >0.6 in the GnRH stimulation test, pelvic ultrasound findings of enlarged uterus and ovaries with multiple follicles (≥4 mm), and accelerated linear growth. Control participants were confirmed to be in the normal stage of growth and development following comprehensive evaluation. All participants were recruited at their parents’ request for assessment of growth and developmental status. Participants were recruited according to the following criteria: i) girls aged 6–10 years and ii) agreement to undergo all required serological and imaging examinations. Exclusion criteria comprised the following: i) age of <6 or >10 years (to exclude the effects of mini-puberty and normal pubertal onset); ii) diagnoses of incomplete, peripheral precocious puberty, or organic CPP due to central lesions; iii) chronic diseases affecting FGF21; iv) genetic syndromes known to perturb FGF21 pathways; v) treatment within the past 3 months with hormones or drugs that interfere with FGF21 metabolism, such as growth hormone or metformin; vi) girls with obesity (BMI standard deviation score > +2 SD for chronological age and sex based on WHO child growth standards); and vii) failure to undergo relevant tests or missing data.

### Data collection

All data were retrospectively extracted from the hospital’s electronic medical record system following ethical approval. Physical development assessments, including height (cm) and weight (kg) measurements, were conducted by two pediatric endocrinologists using standardized instruments. These measurements were then used to calculate BMI and BMI Z-scores. Bone age (BA) was assessed according to the Greulich–Pyle method and interpreted independently by two radiologists. For each participant, Tanner staging (assessed by two pediatric endocrinologists) and BA interpretation (evaluated by two radiologists) were performed during a single clinical visit using standardized tools and imaging. In cases where disagreements arose in Tanner staging or BA interpretation, a third senior physician with subspecialty training in pediatric growth and development – who was blinded to the initial assessments, made the final determination. If the third physician’s evaluation differed from both initial assessors, their judgment was accepted as the final result. Pelvic ultrasonography was performed using a GE Voluson 730 machine (7.5 MHz probe) to measure uterine volume (length × width × anteroposterior diameter × 0.523) and bilateral ovarian volume and to count follicles with a diameter >4 mm.

All enrolled children underwent venipuncture for blood testing at 08:00 h on their first visit, with a concurrent GnRH stimulation test performed to determine eligibility according to CPP diagnostic criteria. All specimens were processed immediately after collection by certified laboratory staff to ensure test validity.

### FGF21 and β-Klotho measurements

FGF21 and β-Klotho levels were determined using the ELISA technique with a DNM-9602 apparatus (manufactured by Beijing Plang Technology Co., Ltd, China). The following parameters were examined: FGF21 (Cat. No. CHE1099; intra-assay: CV < 10%; inter-assay: CV < 12%; sensitivity 15 pg/mL) and β-Klotho (Cat. No. KGC8601-96; intra-assay: CV < 10%; inter-assay: CV < 12%; sensitivity 38 ng/L).

### Statistical analysis

Statistical analysis was conducted using R Studio software. For quantitative data, non-normally distributed variables were presented as medians with interquartile range (M (Q1, Q3)), whereas normally distributed variables were presented as mean ± standard deviation (SD). Categorical data were compared using the chi-square test. For comparisons between groups, an independent-sample *t*-test was used for the normally distributed data. The Wilcoxon rank-sum test was used for comparing data that did not follow a normal distribution. Pearson correlation analysis was performed to evaluate the linear correlations between continuous variables (including FGF21, β-Klotho levels, and clinical indicators such as bone age and hormone levels). The correlation coefficient (*r*) and *P* value were calculated. Diagnostic ability was assessed using receiver operating characteristic (ROC) curves to compare the diagnostic efficacy of FGF21 and β-Klotho in the context of CPP in girls. For the combined ROC analysis of FGF21 and β-Klotho, both biomarkers were entered into binary logistic regression to calculate a unified predicted probability for each subject. The ROC curve and area under the curve (AUC) were generated based on this combined probability score. A two-sided *P*-value of <0.05 was considered statistically significant.

## Results

### Clinical and biochemical characteristics

Between the CPP and control groups, there were no significant differences in age (7.93 ± 0.91 years vs 8.11 ± 1.05 years, *P* > 0.05) or BMI-SDS (−0.51 ± 1.18 vs −0.56 ± 1.31, *P* > 0.05). However, significant differences were observed in height (139.47 ± 7.67 cm vs 127.74 ± 7.75 cm, *P* < 0.001), weight (32.90 (29.00, 40.50) kg vs 25.30 (22.25, 28.50) kg, *P* < 0.001), BA (11.00 (9.00, 11.50) vs 8.00 (7.00, 8.50), *P* < 0.001), BMI (17.67 ± 2.37 kg/m^2^ vs 15.50 ± 1.72 kg/m^2^, *P* < 0.001), and height-SDS (0.92 (0.13, 1.56) vs 0.11 (−0.49, 0.79), *P* < 0.05), as shown in Table 1.

**Table 1:** Clinical and biochemical characteristics.

	CPP group	Control group	*P*
Age (year)	8.05 (6.92, 9.10)	8.08 (7.42, 8.54)	0.369
Height (cm)	139.47 ± 7.67	127.74 ± 7.75	<0.001*
Weight (kg)	32.90 (29.00, 40.50)	25.30 (22.25, 28.50)	<0.001*
BMI (kg/m^2^)	17.67 ± 2.37	15.50 ± 1.72	<0.001*
BMI SDS	−0.56 ± 1.31	−0.51 ± 1.18	0.827
Height SDS	0.92 (0.13, 1.56)	0.11 (−0.49, 0.79)	<0.001*
BA	11.00 (9.00, 11.50)	8.00 (7.00, 8.50)	<0.001*
BAI	1.28 ± 1.25	0.99 ± 0.12	<0.001*
Uterine length (mm)	28.00 (24.00, 33.00)	19.00 (17.50, 22.00)	<0.001*
Uterine volume (cm^3^)	10.35 (6.01, 18.81)	2.17 (1.85, 2.74)	<0.001*
Ovarian length (mm)	26.50 (24.00, 29.00)	24.00 (22.00, 25.50)	<0.001*
Ovarian volume (cm^3^)	5.54 (3.96, 9.74)	3.67 (2.80, 4.90)	<0.001*

BA, bone age; BAI, bone age index.

BAI was calculated as the ratio of bone age (BA), assessed using the Greulich–Pyle method, to chronological age (CA), using the formula: BAI = BA (years)/CA (years).

* Indicates statistical significance (*P* < 0.05).

### Comparison of FGF21 and β-Klotho between the two groups

There were significant differences in serum FGF21 (139.94 (124.63, 150.07) pg/mL vs 178.08 (154.00, 233.31) pg/mL, *P* < 0.001) and β-Klotho (5,910.68 (3,728.42, 6,379.34) pg/mL vs 7,810.63 (5,904.74, 10,381.94) pg/mL, *P* < 0.001) levels between the two groups. Girls in the CPP group had lower serum levels of FGF21 and β-Klotho than those in the control group, as shown in Table 2.

**Table 2:** Comparison of FGF21 and β-Klotho among two groups.

	CPP group	Control group	*P*
FGF21 (pg/mL)	139.94 (124.63, 150.07)	178.08 (154.00, 233.31)	<0.001*
β-Klotho (pg/mL)	5,910.68 (3,728.42, 6,379.34)	7,810.63 (5,904.74, 10,381.94)	<0.001*
SHBG (ng/mL)	134.97 (110.34, 146.73)	111.66 (68.40, 121.89)	<0.001*
Leptin (pg/mL)	1,793.21 ± 1,238.28	1,979.38 ± 1,240.08	0.443
IGF1 (ng/mL)	329.79 (295.80, 393.10)	204.43 (192.21, 237.46)	<0.001*
E2 (pg/mL)	33.37 (14.97, 40.88)	5 (5, 5)	<0.001*
25 (OH) D (ng/mL)	19.75 ± 6.02	21.44 ± 4.22	0.102

* Indicates statistical significance (*P* < 0.05).

### Correlations between FGF21, β-Klotho, and other clinical parameters

FGF21 levels were positively correlated with β-Klotho and 25(OH)D levels but negatively correlated with anthropometric and developmental parameters, including height, weight, BMI, BA, bone age index (BAI), IGF1, uterine length, uterine volume, and ovarian volume. Furthermore, β-Klotho levels were also negatively associated with sex hormone-binding globulin (SHBG), BA, BAI, IGF1, uterine length, and uterine volume, as shown in Tables 3, 4, and 5.

**Table 3:** Correlation between FGF21, β-Klotho, and other serological parameters.

		β-Klotho	SHBG1	Leptin	FGF21	IGF1	E2	25 (OH) D
β-Klotho	r	/	−0.533	0.043	0.236	−0.370	−0.441	0.066
	*P*	/	<0.001*	0.663	0.015*	<0.001*	<0.001*	0.505
SHBG1	r	−0.533	/	−0.115	−0.086	0.288	0.387	−0.169
	*P*	<0.001*	/	0.243	0.384	0.003*	<0.001*	0.084
Leptin	r	0.043	−0.115	/	0.109	0.095	−0.033	0.227
	*P*	0.663	0.243	/	0.27	0.345	0.737	0.02*
FGF21	r	0.236	−0.086	0.109	/	−0.573	−0.482	0.312
	*P*	0.015*	0.384	0.27	/	<0.001*	<0.001*	0.001*
IGF1	r	−0.370	0.288	0.095	−0.573	/	−0.482	−0.027
	*P*	<0.001*	0.003*	0.345	<0.001*	/	<0.001*	0.79
E2	r	−0.441	0.387	−0.033	−0.482	0.753	/	−0.279
	*P*	<0.001*	<0.001*	0.737	<0.001*	<0.001*	/	0.004*
25 (OH) D	r	0.066	−0.169	0.227	0.312	−0.027	−0.279	/
	*P*	0.505	0.084	0.02*	0.001*	0.79	0.004	/

* Indicates statistical significance (*P* < 0.05).

**Table 4:** Correlation between FGF21, β-Klotho, age, uterus, and ovary.

		Age	Uterine length	Uterine volume	Ovarian length	Ovarian volume
β-Klotho	r	−0.049	−0.298	−0.252	0.021	−0.107
	*P*	0.619	0.002*	0.009*	0.829	0.276
SHBG1	r	0.035	0.413	0.383	0.172	0.198
	*P*	0.723	<0.001*	<0.001*	0.08	0.043*
Leptin	r	−0.160	−0.124	−0.032	0.136	0.12
	*P*	0.104	0.208	0.743	0.166	0.222
FGF21	r	−0.164	−0.348	−0.382	0.014	−0.235
	*P*	0.095	<0.001*	<0.001*	0.889	0.016*
IGF1	r	0.139	0.637	0.789	0.305	0.478
	*P*	0.163	<0.001*	<0.001*	0.002*	<0.001*
E2	r	0.071	0.759	0.813	0.412	0.510
	*P*	0.473	<0.001*	<0.001*	<0.001*	<0.001*
25 (OH) D	r	−0.276	−0.317	−0.208	−0.013	−0.165
	*P*	0.004*	0.001*	0.033*	0.898	0.093

* Indicates statistical significance (*P* < 0.05).

**Table 5:** Correlation between FGF21, β-Klotho, and other anthropometric parameters.

		Height	Weight	BMI	BA	Height SDS	BMI SDS	BAI
β-Klotho	r	−0.187	−0.174	−0.079	−0.313	−0.108	0.166	−0.390
	*P*	0.056	0.076	0.425	0.001*	0.272	0.09	<0.001*
SHBG1	r	0.295	0.328	0.276	0.491	0.03	−0.04	0.566
	*P*	0.002*	0.001*	0.004*	<0.001*	0.763	0.686	<0.001*
Leptin	r	−0.245	−0.273	−0.219	−0.198	−0.194	−0.171	−0.12
	*P*	0.012*	0.005*	0.025*	0.043*	0.047*	0.081	0.223
FGF21	r	−0.319	−0.381	−0.324	−0.474	−0.184	0.042	−0.395
	*P*	<0.001*	<0.001*	0.001*	<0.001*	0.06	0.667	<0.001*
IGF1	r	0.569	0.524	0.351	0.723	0.407	−0.218	0.676
	*P*	<0.001*	<0.001*	<0.001*	<0.001*	<0.001*	0.028*	<0.001*
E2	r	0.519	0.533	0.371	0.672	0.414	−0.075	0.705
	*P*	<0.001*	<0.001*	<0.001*	<0.001*	<0.001*	0.445	<0.001*
25 (OH) D	r	−0.346	−0.318	−0.154	−0.204	−0.015	−0.066	−0.044
	*P*	<0.001*	0.001*	0.117	0.037*	0.88	0.5	0.656

BA, bone age; BAI, bone age index. BAI was calculated as the ratio of BA, assessed using the Greulich–Pyle method, to chronological age (CA), using the formula: BAI = BA (years)/CA (years).

* Indicates statistical significance (*P* < 0.05).

### FGF21, β-klotho levels, and CPP proportion

According to interquartile ranges, FGF21 levels were categorized into Q1 (<132.9), Q2 (132.9–150.6), Q3 (150.6–180.9), and Q4 (>180.9), while β-Klotho levels were grouped into Q1 (<4,585.5), Q2 (4,585.5–6,303.8), Q3 (6,303.8–7,783.4), and Q4 (>7,783.4). Stratification based on these quartiles revealed that the proportion of girls with CPP progressively decreased with ascending quartile levels of both biomarkers (both *P* < 0.001), as shown in Table 6.

**Table 6:** FGF21, β-Klotho levels, and CPP proportion.

	Control group	CPP group	*χ* ^2^	*P*
FGF21, pg/mL				
Q1 (<132.9)	8	18	44.702	<0.001*
Q2 (132.9–150.6)	3	24		
Q3 (150.6–180.9)	16	12		
Q4 (>180.9)	24	0		
β-Klotho, pg/mL				
Q1 (<4,585.5)	9	17	37.127	<0.001*
Q2 (4,585.5–6,303.8)	7	20		
Q3 (6,303.8–7,783.4)	9	17		
Q4 (>7,783.4)	26	0		

* Indicates statistical significance (*P* < 0.05).

### ROC analysis

**Figure 1:**
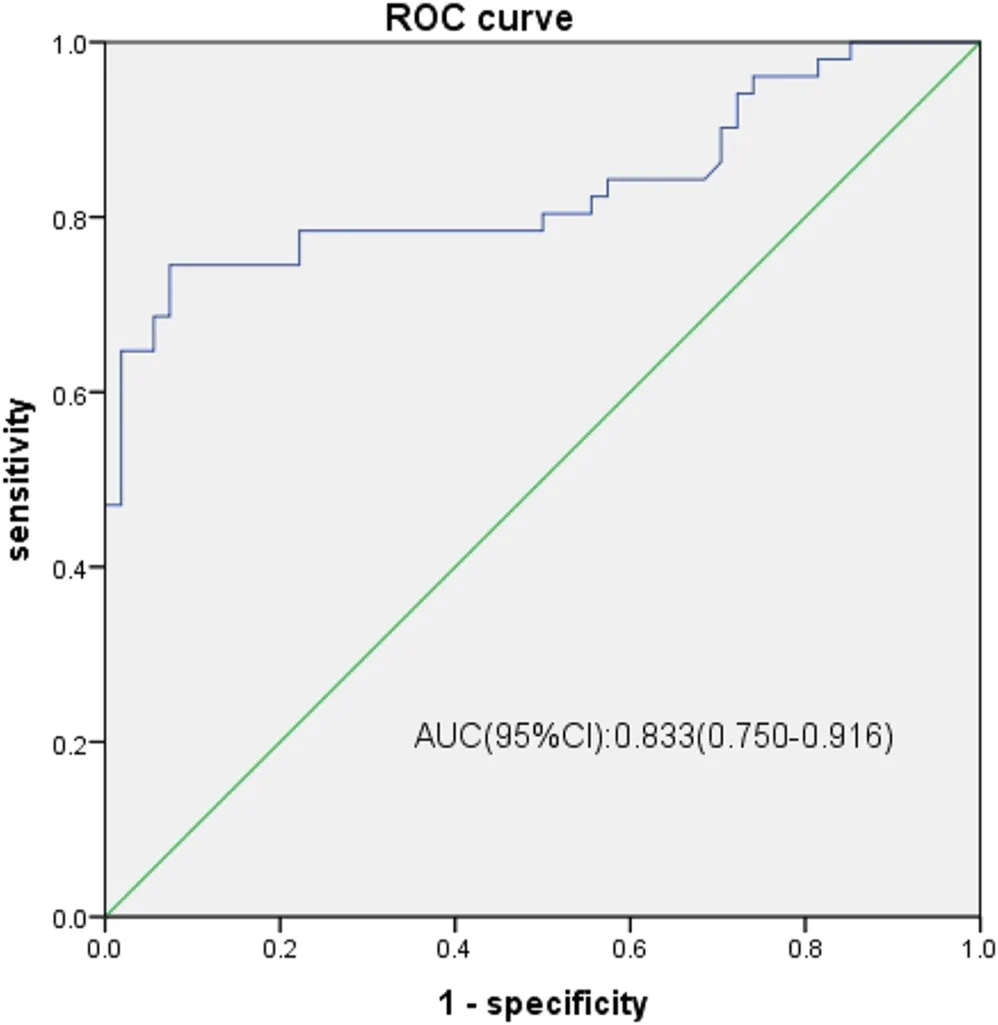
ROC curve for evaluating the predictive ability of FGF21 for CPP. AUC, area under the curve; 95% CI, 95% confidence interval; ROC, receiver operating characteristic.

**Figure 2:**
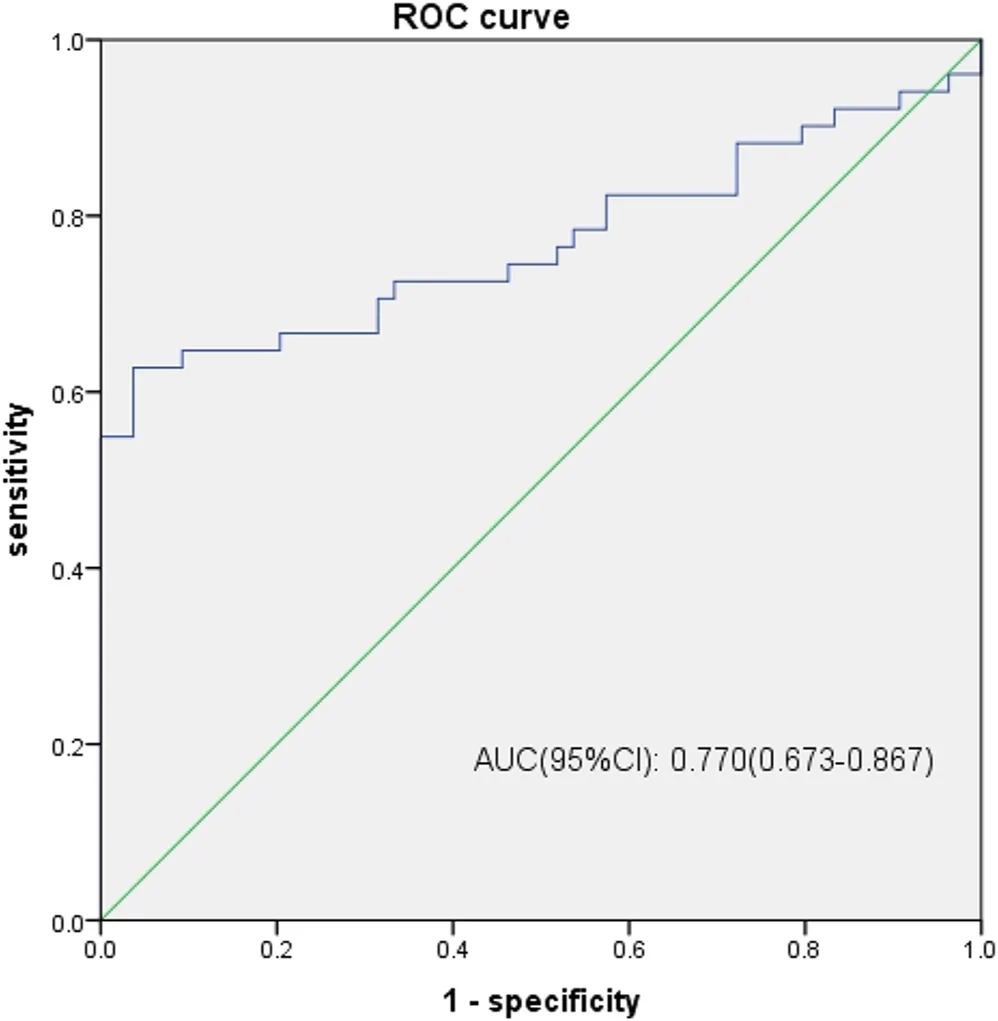
ROC curve for evaluating the predictive ability of β-Klotho for CPP. AUC, area under the curve; 95% CI, 95% confidence interval; ROC, receiver operating characteristic.

**Figure 3:**
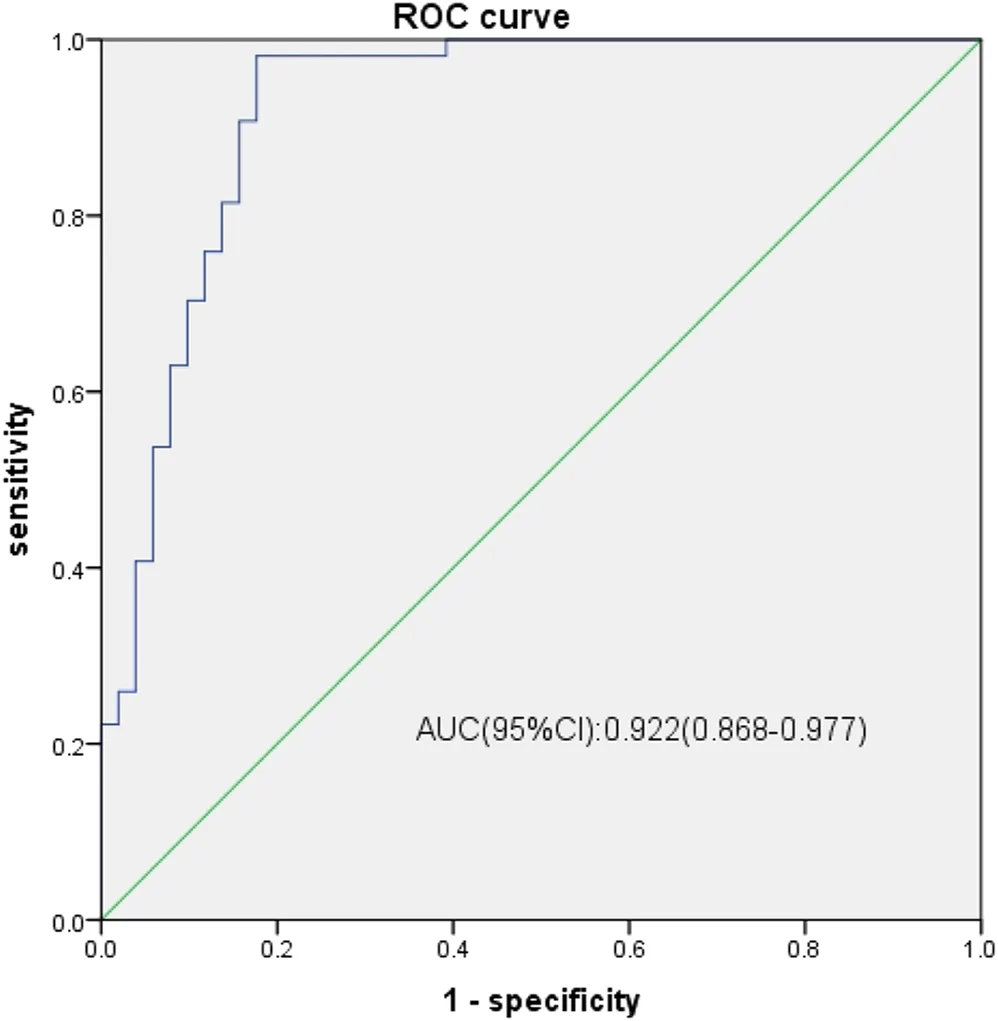
ROC curve for evaluating the predictive ability of FGF21 combined with β-Klotho for CPP. AUC, area under the curve; 95% CI, 95% confidence interval; ROC, receiver operating characteristic.

ROC curve analysis revealed that the AUCs of FGF21 and β-Klotho levels for distinguishing CPP from controls were 0.833 (cut-off value: 156.27 pg/mL; sensitivity: 74.5%; specificity: 92.6%) and 0.770 (cut-off value: 7,058.18 pg/mL; sensitivity: 62.7%; specificity: 92.3%), respectively (as shown in Figs 1, 2, and 3). When these two indicators were combined, the AUC was 0.922 (as shown in Fig. 3, Table 7).

**Table 7:** ROC curve for evaluating the predictive ability of FGF21 and β-Klotho for CPP.

Characteristic	AUC	95% CI
FGF21	0.833	0.750–0.916
β-Klotho	0.770	0.673–0.867
FGF21 combined with β-Klotho	0.922	0.868–0.977

## Discussion

Our results demonstrated reduced serum FGF21 levels in girls with CPP compared with healthy controls. Previous studies have shown that FGF21 negatively regulates kisspeptin expression (7). FGF21 can cross the blood–brain barrier (23), enabling it to exert direct regulatory effects within the central nervous system. Recent mouse model studies evidence (24, 25, 26) indicate that within the brain, FGF21 suppresses the activity of anteroventral periventricular (AVPV) kisspeptin neurons. This central action impairs induction of the preovulatory LH surge, dampens overall HPGA activity, and consequently leads to anovulation and a prolonged diestrus phase. Combined with our results, these findings suggest that reduced levels of FGF21 and β-Klotho may lead to increased kisspeptin expression, thereby contributing to the onset of CPP.

As an indispensable co-receptor for FGF21, β-Klotho is a central component of FGF receptor-mediated signal transduction (10). In contrast to the broad tissue distribution of FGF receptors, β-Klotho expression is restricted to a select subset of tissues, including the liver, white and brown adipose tissues, and specific hypothalamic regions (10, 27), the latter being critical for the hormone’s central reproductive effects. Specifically, prior studies (7) have established that FGF21, acting as a key mediator of the liver–brain axis, binds to β-Klotho in the hypothalamic suprachiasmatic nucleus under conditions of energy deficiency (e.g. fasting), thereby suppressing the kisspeptin–GnRH pathway to modulate reproductive timing. Animal studies confirm (10) a unidirectional expression linkage between FGF21 and β-Klotho: β-Klotho abundance determines FGF21 bioactivity, and β-Klotho deficiency triggers compensatory FGF21 upregulation, whereas excess FGF21 exerts no feedback effect on β-Klotho expression. Consistent with this mechanistic framework, our study found significantly lower serum β-Klotho levels in girls with CPP than in age-matched controls. Collectively, these observations provide strong mechanistic support for our core finding: the concurrent reduction in serum FGF21 and β-Klotho levels in girls with CPP may impair inhibitory regulation of the kisspeptin–GnRH axis, thereby contributing to premature activation of the HPGA and the onset of CPP.

Animal model evidence (28) has demonstrated that alternate-day fasting not only delays pubertal development in normal-weight mice but also prevents diet-induced obesity and associated precocious puberty. Furthermore, clinical studies (29) have consistently identified childhood obesity as a key contributor to earlier pubertal onset and elevated estradiol levels in girls. Notably, fasting is well-documented to increase serum FGF21 concentrations (30), whereas obesity is known to induce FGF21 resistance (31), a state that impairs the hormone’s biological activity. Collectively, these observations support our proposition that perturbations in circulating FGF21 (either reduced levels or impaired functionality) may play a role in the pathogenesis of CPP in girls, a hypothesis further supported by the key findings of the present study.

In addition, correlation analysis revealed that FGF21 levels were inversely associated with circulating IGF1 (32, 33). In this study, a negative correlation was observed between IGF1 and FGF21 levels, consistent with previous reports. However, no significant association was found between FGF21 and leptin. To mitigate the potential confounding effect of FGF21 resistance resulting from obesity, hyperlipidemia, and diabetes (34, 35), children with those conditions were excluded from the current study. The absence of correlation strongly suggests that in normal-weight girls with CPP, regulation of FGF21 may operate independently of the classical adipokine signal leptin and could instead be driven by other metabolic pathways or sex hormones themselves. However, this non-significant finding could also be attributable to insufficient statistical power, clinical heterogeneity across subjects, and residual unadjusted confounders. Additional adequately powered studies are required to elucidate their exact relationship.

The observation of elevated SHBG levels in our CPP cohort contrasts with the decreased levels reported in previous studies (36), suggesting that the mechanisms driving CPP may be more diverse than previously recognized. In our cohort, however, an alternative pathway may be operative. We speculate that reduced FGF21 levels might disinhibit the HPGA, contributing to the onset of puberty. The consequent increase in E2 could, in turn, stimulate SHBG production (37).

Although vitamin D deficiency has been proposed as a contributing factor in pubertal processes (38), the present study observed no significant association between vitamin D levels and CPP, despite lower mean levels in the CPP group. Given the limited sample size, this preliminary observation requires validation in larger-scale studies.

This study has several limitations that warrant acknowledgment. First, because CPP diagnosis was partially based on elevated gonadotropin levels, no statistical comparison of hormonal profiles was performed among participants. This limitation will be addressed in subsequent investigations of rapidly progressive CPP incorporating comprehensive hormonal analyses. Our research primarily focused on FGF21/β-Klotho associations with CPP rather than diagnostic efficacy; in addition, because CPP diagnosis was established using sex hormone profiles, comparative analyses of diagnostic sensitivity and specificity between FGF21/β-Klotho and peak gonadotropin levels were not conducted. Second, β-Klotho levels were not assessed in male participants, leaving unresolved whether a similar expression pattern exists in boys with CPP. Third, FGF21 and β-Klotho levels after GnRH agonist therapy were not measured, and such assessments would provide critical insights into their mechanistic roles in CPP pathophysiology.

## Conclusion

Our study provides preliminary evidence that serum levels of FGF21 are decreased in girls with CPP. Furthermore, we found an association between FGF21 levels and the timing of pubertal onset, providing a basis for the clinical diagnosis and potential therapeutic exploration of CPP. However, the causal relationship between FGF21 and CPP, as well as the underlying molecular mechanisms, remains to be further investigated.

## Declaration of interest

The authors declare that there is no conflict of interest that could be perceived as prejudicing the impartiality of the work reported.

## Funding

This work did not receive any specific grant from any funding agency in the public, commercial, or not-for-profit sectors.

## Author contribution statement

XH conceived and designed the study; acquired, analyzed, and interpreted the data; drafted and critically revised the manuscript for important intellectual content; and provided final approval of the version to be published. JW collected and analyzed the data; critically revised the manuscript for important intellectual content; and provided final approval of the version to be published. RZ conceived and designed the study; interpreted the data; critically revised the manuscript for important intellectual content; and provided final approval of the version to be published. MS collected and analyzed data and critically revised the manuscript for important intellectual content. YC analyzed data; critically revised the manuscript; and provided final approval of the version to be published. All authors agreed to be accountable for all aspects of the work.

## Data availability

Data are not publicly available due to ethical reasons. Further inquiries can be directed to the corresponding author.

## AI disclosure

No generative artificial intelligence (AI) tools were used in the preparation of the written or visual content of this manuscript.

## Statement of ethics

Ethical approval for this study was granted by the Hospital Ethics Committee (Approval No. 2025-225; April 8, 2025). According to the ethical approval provided by the aforementioned ethics committee, written informed consent was not required in accordance with current national guidelines and regulations and the requirements of the World Medical Association’s ‘Helsinki Declaration’. One physician from the research team met with all participants and their parents to obtain informed consent from the parents. Relevant information of each participant’s family was recorded.
